# Knowledge, attitudes, and practices of cervical Cancer screening among HIV-positive and HIV-negative women participating in human papillomavirus screening in rural Zimbabwe

**DOI:** 10.1186/s12905-020-01017-2

**Published:** 2020-07-25

**Authors:** Megan Fitzpatrick, Mythili P. Pathipati, Kathy McCarty, Anat Rosenthal, David Katzenstein, Z. M. Chirenje, Benjamin Pinsky

**Affiliations:** 1 Department of Pathology, University of Wisconsin, 600 Highlands Ave, Madison, WI USA; 2 Department of Internal Medicine, Massachusetts General Hospital, 55 Fruit Street, Boston, MA 02114 USA; 3 Chidamoyo Christian Hospital, P.O. Box 330, Karoi, Zimbabwe; 4 Department of Health Systems Management, Ben-Gurion University of the Negev, P.O.B. 653, 8410501 Beer-Sheva, Israel; 5 Biomedical Research and Training Institute of Zimbabwe, 10 Seagrave Rd, Mount Pleasant, Harare, Zimbabwe; 6 Department of Obstetrics and Gynecology, University of Zimbabwe College of Health Science, Harare, Zimbabwe; 7 Department of Pathology, Stanford Hospitals and Clinics, 300 Pasteur Drive, Stanford, CA USA

**Keywords:** Human papillomavirus, Knowledge, attitudes and practices, Cervical Cancer, Cervical Cancer screening, Human immunodeficiency virus, Zimbabwe

## Abstract

**Background:**

Women in low- and middle-income countries are at the highest risk of cervical cancer yet have limited access to and participation in cervical cancer screening programs. Integrating self-collected, community-based screening offers a potential primary screening method in areas of limited resources. In this paper, we present a study evaluating knowledge, attitudes, and practices of cervical cancer and Human Papilloma Virus (HPV) in rural Zimbabwe.

**Methods:**

We performed a community-based cross-sectional knowledge, attitudes and practices of HPV and cervical cancer study in rural Zimbabwe from January 2017–May 2017. Women were selected for the study via random number generation from complete lists of inhabitants in the study area if they satisfied the inclusion criteria (≥30-years-old, ≤65-years-old, not pregnant, intact uterus). If selected, they participated in a 19-question structured knowledge, attitudes and practices survey. The questionnaire included questions on demographics, education, knowledge of HPV, cervical cancer, and risk factors. Chi-squared tests were evaluated comparing knowledge, attitudes and practices relating to HPV and cervical cancer screening with actual infection with HPV. Women were also offered a voluntary HIV and self-collected HPV screening.

**Results:**

Six hundred seventy-nine women were included in the knowledge, attitudes and practices survey. Most women (81%) had heard of cervical cancer while the majority had not heard of HPV (12%). The number of women that had been screened previously for cervical cancer was low (5%)**.** There were no significant differences between and within groups regarding knowledge of cervical cancer and actual overall infection with HR-HPV, HPV 16, and HPV 18/45 test results.

**Conclusions:**

Most women in rural Zimbabwe have heard of cervical cancer, but the number that had been screened was low**.** Extending existing outreach services to include cervical cancer screening, potentially including HPV screening, should include cervical cancer/HPV education and screening triage. This approach would serve to bridge the gap between knowledge and screening availability to address some of the barriers to cervical cancer care still affecting women in many regions of the world.

## Background

Cervical cancer disproportionately affects women in low- and middle-income countries (LMICs) where it is the second most common cancer in women. LMICs account for 84% of new cases worldwide with 8 out of 10 cases occurring in Sub-Saharan Africa [1].

Malawi, Mozambique, Comoros, and Zimbabwe have the highest burden of cervical cancer in Sub-Saharan Africa [2]. In Zimbabwe, cervical cancer is the most common cancer among women and the leading cause of female cancer deaths [3]. Approximately 2270 women are diagnosed with cervical cancer in Zimbabwe and 1451 women die from cervical cancer each year [3]. Structural inequities contribute to a high burden of cervical cancer in Zimbabwe including inadequate screening infrastructure, facilities and personnel. Rural women are at particularly high risk of cervical cancer due to fear of participation, patriarchal social structures, stigma around cancer and reproductive health, male dominated medical providers in rural areas, and disproportionate poverty. The number of women living in poverty in rural areas has risen 50% in the last two decades in what is referred to as the “feminization of poverty” [4]. Accessing these women in screening programs is critical to successful disease eradication.

Human Papillomavirus (HPV), a sexually transmitted infection, is the causative infection of > 99% of cervical cancers [5]. Of note, HIV prevalence is important as well because HPV is an opportunistic infection in the setting of HIV. In 2016, 1.3 million people were estimated to be living with HIV in Zimbabwe with 40,000 new infections [6]. While 14% of Zimbabwean adults age 15–49 are HIV positive, women are disproportionately infected (17% among women vs. 11% among men) [7].

HPV detection and vaccination are therefore standard elements of screening and prevention programs, including in protocols recommended by the World Health Organization (WHO). Pre-cancerous lesions can be detected 10+ years before cervical cancer develops. An estimated 90% of cervical cancer could be prevented if all women were offered and participated in high quality cytological screening programs [8]. Detection of HPV may serve as a viable objective triage step, especially due to the possibility of self-collection, which could improve participation and access for women, while decreasing the burden on healthcare providers in overburdened low resource areas.

Robust screening programs are still lacking in the regions that are most affected. For instance, Zimbabwe’s Ministry of Health and Child Welfare (MOHCW) has expanded prevention of cervical cancer by scaling up cervical screening services as part of the government’s National Health Strategy. With financial support from the World Health Organization and United Nations Population Fund, the MOHCW has initiated a national program for cervical cancer screening and management using visual inspection with acetic acid plus cervicography (VIAC) [9].

Unfortunately, limited VIAC-trained healthcare providers and resources are available and consequently only a small subset of women will be screened in their lifetime (9.4% in urban areas, and 5% in rural areas of Zimbabwe) [10]. Recently, the MOHCW has implemented HPV vaccination among young women to further the efforts at disease control, with support from the Global Alliance on Vaccine Initiative (GAVI). In the meantime, continued screening is critical to prevent/detect precancerous and cancerous lesions among those already infected.

While establishing a successful screening program is a multi-faceted challenge, one key element is the buy-in and awareness of the population that needs to be screened. In the case of cervical cancer, women’s knowledge has been shown to affect subsequent health seeking behaviors [11], but limited work has been done to characterize the knowledge, attitudes, and practices (KAP) associated with cervical cancer among women in high-risk regions.

In this paper, we therefore evaluate these factors in relation to cervical cancer and HPV in rural Zimbabwe in a community-based screening program. We combined self-collected community-based HPV collection with a KAP survey across 16 sites in rural village centers in Wards 13/15 in Hurungwe district of Zimbabwe. We compared these survey results to actual infections with HPV and HIV in the population studied to understand how education, sexual practices, and knowledge of cervical cancer affect a woman’s probability of being infected with HPV or HIV.

## Methods

We conducted a randomized community-based cross-sectional study in in rural northwestern Zimbabwe (Hurungwe district in Mashonaland West) with the study area defined as Ward 13/15 (the approximate catchment area of Chidamoyo Christian Hospital) beginning in mid-January 2017 to mid-May 2017 [12, 13]. Three months prior to sample collection (2016), all community health workers from Ward 13 & 15 were provided training on HPV and cervical cancer and community screening methodologies and asked to submit complete lists of all women from 30 to 65 in their villages.

A total of 3108 women aged 30–65 were identified in Ward 13/15. The names and communities of the women were entered in an excel spreadsheet and a random number generator was used to select potential participants in all villages. Nine hundred forty-six women were invited to participate in the study.

The women participated in a 19-question structured knowledge, attitudes and practices survey from January 2017–May 2017 in rural northwestern Zimbabwe (Hurungwe district in Mashonaland West), previously validated in other similar studies in urban Zimbabwe [14]. The questionnaire included questions on demographics, education, knowledge of HPV, cervical cancer, and risk factors.

In addition, women were asked to participate in village-based self-collected HR-HPV testing in conjunction with existing community outreach models for the distribution of antiretroviral therapy (ART) and the World Health Organization Expanded Program on Immunization (EPI) outreach in villages in rural Zimbabwe from January 2017 through May 2017. The results of self-collection HR-HPV are described in more detail in a prior study [12]. We did however use this data to contextualize the results from the knowledge, attitudes, and practices survey and to determine if knowledge about cervical cancer affected actual infection with HPV.

HIV status was also determined in the prior study and used in our study to determine whether HIV infection had any impact on knowledge, attitudes, and practices regarding cervical cancer [12]. HIV serologic testing was performed by Ministry of Health certified HIV counselors with the Ministry-provided 3rd generation Alere Determine HIV-1/2 test (Alere/Abbott, Lake Bluff, Illinois, U.S.), a qualitative immunoassay for the detection of antibodies to HIV-1 and HIV2 [12].

Village health workers personally invited eligible women to attend collection sites in their villages on assigned dates. If women were not present at the outreach site on the day of collection, they were contacted by the village health worker again and invited to the next nearest site and/or visited at their homes. Non-attendees were approached at home to determine if they were refusing testing, and the reason for refusal to obtain information for potential refusal bias [12].

All women were offered a bar of soap (<$1USD value) as an incentive even if they were disqualified (due to age or pregnancy) [12]. Pregnant women and women under 30 years of age were excluded because they are known to have a higher prevalence of HR-HPV infections with complex clearance cycles not observed in non-pregnant women. Women under 30 years old have higher prevalence rates of HR-HPV infection, but lower cervical intraepithelial neoplasia (CIN) and cervical cancer detection rates.

Cervicovaginal swab self-collection was performed in the community during scheduled outreach visits for provision of ART medications and childhood vaccines, and HR-HPV testing was performed at the community hospital.

### Statistical methods

All analyses were performed with STATA version 15 (*Stata Statistical Software: Release 15.* College Station, TX: StatCorp LLC). Descriptive statistics were used to analyze the demographic and knowledge, attitudes, and practices relating to cervical cancer based on a previously validated KAP survey. Chi-squared tests were evaluated comparing women these factors of knowledge, attitudes and practices with actual infection with HPV and in patients with HIV. Significance was determined at *p* < 0.05 for all statistical tests.

### Ethical consent

Ethical approval was granted by Stanford University (#37975), University of Zimbabwe (JREC 221/16), the Medical Research Council of Zimbabwe (MRCZ/A/2128), and the Research Council of Zimbabwe (No. 02921). In addition, the Provincial and District Medical Officers were notified, as well as headmen and villages during community meetings, after sensitization via training of community health workers prior to data collection. Women were informed that their participation was voluntary, they could withdraw at any time, that we would offer to test for HIV but they could refuse this testing or refuse to be notified of their result, and that all information regarding their HIV and HPV status would be kept confidential. Most women wanted to know their HIV results. Inclusion took place after individual informed consent signed electronically with a paper copy given to the participant informed consent (signature or witnessed thumbprint) was obtained from all participants prior to enrolment. Eligible women were interviewed by trained research data collectors on the research team using an electronic questionnaire to collect information on sociodemographic and reproductive information.

## Results

### Demographics

The study participants were between 30 and 65 years with a mean age of 43 years. Six hundred seventy-nine women were included in the survey. As shown in Table 1, the ages of the participants were distributed as follows: the majority (44.5%) were in the age group < 40 years with 27.4% of participants in the 40–50 age range and 28.1% in the > = 50 age group. Most of the participants had a grade 7 level education (49.4%) with 24% never having attended school, 26% obtaining an Ordinary Level education (the first phase of secondary education in Zimbabwe) and less than 1% obtaining an Advanced level education (the second phase of secondary education that is required for entry into universities in Zimbabwe). The majority, 66.8%, of participants had their first pregnancy between age 15 and 20 while 35% had their first pregnancy between 20 and 30 years, 15.7% had their first pregnancy at age > =30 years, and 2.5% and age < 15 years.

**Table 1:** Demographics Characteristic of Survey Participants Sociodemographic characteristics of women who participated in the study

		Count	%
Age	< 40	302	44.5%
	40–50	186	27.4%
	> = 50	191	28.1%
Level of Education	Did not attend	163	24%
	O′ Level	177	26%
	A’ Level	3	0.44%
	Grade 7	336	49.4%
Age of First Pregnancy	< 15	14	2.4%
	15–20	356	61.81%
	20–30	201	34.90%
	> = 30	106	15.65%

### Knowledge and attitudes of cervical Cancer

Most women (81.2%) had heard of cervical cancer while the majority had not heard of HPV (11.7%). Many heard about cervical cancer from a Village Health Worker (26.5%), the radio (21.7%), hospital staff (21.2%), or a friend (22.8%). In spite of this broad awareness, most women had never received screening (4.9%). Table 2 summarizes knowledge across different demographic categories.

**Table 2:** Knowledge of Cervical Cancer by Demographics

	Age			Education				Age of First Pregnancy			
	< 40	40–50	> = 50	Did not attend	O′ Level	A’ Level	Grade 7	< 15	15–20	20–30	> = 30
Have you heard of cervical cancer?											
Yes	242 (80.4%)	156 (84.32%)	151 (79.47%)	120 (74.04%)	148 (83.62%)	2 (66.67%)	279 (83.53%)	9 (64.29%)	295 (83.10%)	154 (76.62%)	15 (14.15%)
No	59 (19.6%)	29 (15.7%)	39 (20.5%)	42 (25.93%)	29 (16.38%)	1 (33.33%)	55 (16.47%)	5 (35.71%)	60 (16.90%)	47 (23.38%)	91 (85.84%)
From Whom Did you heard about cervical cancer?											
Family Member	17 (7.08%)	8 (5.26%)	17 (11.56%)	6 (5.17%)	12 (8.16%)	0 (0%)	24 (8.67%)	3 (33.33%)	20 (6.99%)	17 (11.11%)	5 (5.49%)
Friend	37 (15.42%)	36 (23.68%)	50 (34.01%)	47 (40.52%)	16 (10.88%)	0 (0%)	60 (21.90%)	0 (0%)	64 (22.38%)	28 (18.30%)	28 (30.7%)
Hospital Staff	55 (22.29%)	36 (23.68%)	23 (15.65%)	17 (14.66%)	35 (23.81%)	0 (0%)	62 (22.63%)	3 (33.33%)	59 (20.63%)	31 (20.26%)	21 (23.07%)
Radio	54 (22.50%)	28 (18.42%)	25 (23.81%)	20 (17.24%)	44 (29.93%)	0 (0%)	53 (19.34%)	1 (11.11%)	63 (22.03%)	37 (24.18%)	16 (17.50%)
Village Health Worker	77 (32.08%)	44 (29.95%)	22 (14.97%)	26 (22.41%)	40 (27.21%)	2 (100%)	75 (27.37%)	2 (22.22%)	80 (27.97%)	40 (26.14%)	21 (23.07%)
Have you heard of HPV/Human Papilloma Virus?											
Yes	34 (11.30%)	22 (11.83%)	23 (12.11%)	16 (9.94%)	23 (13%)	0 (0%)	40 (11.90%)	2 (14.29%)	46 (12.92%)	23 (11.44%)	98 (92.4%)
No	267 (88.70%)	164 (88.17%)	167 (87.89%)	145 (90.06%)	154 (87%)	2 (100%)	269 (88.10%)	12 (85.71%)	310 (87.08%)	178 (88.56%)	8 (7.54%)
Please tell me whether you agree or disagree: Cervical cancer can be prevented.											
Yes	268 (89.04%)	165 (88.71%)	153 (80.10%)	121 (74.69%)	159 (89.83%)	3 (100%)	303 (90.18%)	13 (92.86%)	308 (86.52%)	169 (84.08%)	96 (89.71%)
No	10 (3.32%)	5 (2.69%)	5 (2.62%)	8 (4.94%)	6 (3.39%)	0 (0%)	6 (1.79%)	0 (0%)	11 (3.09%)	8 (3.98%)	1 (0.93%)
I don’t know	23 (7.64%)	16 (8.60%)	33 (17.28%)	33 (20.37%)	12 (6.78%)	0 (0%)	27 (8.04%)	1 (7.14%)	37 (10.39%)	24 (11.94%)	10 (9.34%)
Have you been tested for HPV or cervical cancer (PAP smear)?											
Yes	12 (3.97%)	13 (6.99%)	8 (4.19%)	6 (3.68%)	10 (5.65%)	0 (0%)	17 (5.06%)	0 (0%)	20 (5.62%)	10 (4.98%)	3 (2.7%)
No	290 (96.03%)	173 (93.01%)	183 (95.81%)	157 (96.32%)	167 (94.35%)	3 (100%)	319 (94.94%)	14 (100%)	336 (94.38%)	191 (95.02%)	105 (97.2%)
Have you been diagnosed with cervical cancer?											
Yes	2 (0.66%)	4 (2.16%)	2 (1.05%)	2 (1.23%)	1 (0.57%)	0 (0%)	5 (1.49%)	0 (0%)	5 (1.14%)	2 (1%)	1 (0.92%)
No	300 (99.24%)	181 (97.84%)	189 (98.95%)	161 (98.77%)	175 (99.43%)	3 (100%)	331 (98.51%)	14 (100%)	350 (98.59%)	199 (99%)	107 (99.07%)
What was your age at first sexual intercourse/sexual debut?											
AVERAGE	18.41	18.71	18.69	16.3	18.89	18.33	17.95	13.46	16.97	20.58	20.78
Have you ever used oral contraceptive pills?											
Yes	245 (81%)	127 (68.28%)	108 (56.84%)	97 (59.88%)	133 (75.14%)	3 (100%)	247 (73.51%)	12 (85.71%)	261 (73.31%)	128 (63.68%)	79 (73.83%)
No	45 (14.90%)	53 (28.49%)	76 (40%)	61 (37.65%)	35 (19.77%)	0 (0%)	78 (23.21%)	2 (14.29%)	80 (22.47%)	64 (31.84%)	28 (26.16%)
Is your husband/partner circumcised?											
Yes	27 (9.41%)	14 (8.14%)	14 (8.59%)	13 (8.97%)	16 (9.58%)	1 (33.33%)	25 (8.14%)	0 s(0%)	34 (10.15%)	19 (9.74%)	2 (2.53%)
No	258 (89.90%)	154 (89.53%)	144 (88.34%)	128 (88.28%)	147 (88.02%)	2 (66.67%)	279 (90.88%)	13 (100%)	297 (88.66%)	171 (87.69%)	75 (94.93%)
I don’t know	2 (0.70%)	4 (2.33%)	5 (3.07%)	4 (2.67%)	4 (2.40%)	0 (0%)	3 (0.98%)	0 (0%)	4 (1.19%)	5 (2.56%)	2 (2.53%)

### HPV status

Table 3 shows the HPV status of the women surveyed broken down by demographics and their answers to the survey questions. There was no significant difference in the HPV test results for women who had heard of cervical cancer (*p* = 0.548), HPV (*p* = 0.507), or for women who knew that cervical cancer could be prevented (*p* = 0.063). In addition, no significant difference existed for women who had been tested for HPV or cervical cancer with a pap smear (*p* = 0.615) or for those who had been diagnosed with cervical cancer (*p* = 0.146). Finally, no significant differences existed in the HPV test results for women who had used oral contraceptive pills (*p* = 0.752). The sample was too small to reliably comment on the significance of HPV tests results for those whose husband/partner was circumcised.

**Table 3:** Knowledge About Cervical Cancer in Relation to Overall HPV

	Positive	Negative
Age		
< 40	59 (19.7%)	239 (80.20%)
40–50	31 (16.94%)	152 (83.06%)
> =50	31 (16%)	162 (83.93%)
Chi-Square/*P*-value	1.295/ 0.523	
Education		
Did not attend	31 (19.14%)	131 (80.86%)
O′ Level	36 (20.57%)	139 (79.4%)
A’ Level	0 (0%)	3 (100%)
Grade 7	54 (16.16%)	280 (83.83%)
Chi-Square/*P*-value	1.809/ 0.613	
Age of First Pregnancy		
< 15	4 (28.5%)	10 (71.43%)
15–20	63 (17.95%)	288 (82.05%)
20–30	39 (19.60%)	160 (80.4%)
> =30	15 (14.29%)	95 (86.36%)
Chi-Square/*P*-value	2.828/ 0.418	
By response to “have you heard of cervical cancer?”		
Yes	95 (17.56%)	446 (82.43%)
No	25 (19.84%)	101 (80.15%)
Chi-Square/*P*-value	0.360/0.548	
By response to “have you heard of HPV/human papilloma virus”		
Yes	16 (20.51%)	62 (79.49%)
No	103 (17.46%)	487 (82.54%)
Chi-Square/*P*-value	0.440/0.507	
Response to: “please tell me whether you agree or disagree: Cervical cancer can be prevented.”		
Yes	97 (16.78%)	481 (83.21%)
No	7 (35%)	13 (65%)
I don’t know	16 (22.54%)	55 (77.46%)
Chi-Square/*P*-value	5.50/ 0.063	
Response to “Against what disease does the HPV vaccine protect?”		
Correct	8 (23%)	27 (77%)
Incorrect	112 (18%)	513 (82%)
Chi-Square/*P*-value	0.543/ 0.461	
Have you been Tested for HPV/Cervical cancer?		
Yes	7 (21.21%)	26 (78.78%)
No	113 (17.74%)	523 (82.10%)
Chi-Square/*P*-value	0.252/0.615	
Have you been diagnosed with cervical cancer?		
Yes	3 (37.50%)	5 (62.50%)
No	117 (17.70%)	544 (82.29%)
Chi-Square/*P*-value	2.105/0.146	
What was your age at first sexual intercourse?		
< 15	8 (34.78%)	15 (65.22%)
15–20	58 (16.29%)	298 (83.70%)
20–30	31 (20.31%)	123 (79.85%)
> =30	24 (17.65%)	117 (82.90%)
Chi-Square/*P*-value	5.67/0.128	
Have you ever used oral contraceptive pills?		
Yes	87 (18.23%)	388 (81.68%)
No	30 (17.24%)	144 (82.75%)
Chi-Square/*P*-value	0.095/ 0.752	
Is your husband/partner circumcised?		
Yes	8 (15.38%)	44 (84.61%)
No	103 (18.69%)	448 (81.30%)
I don’t know	9 (81.82%)	2 (18.18%)
Chi-Square/*P*-value	27.95/< 0.00001	
Years on ART		
> 2 years	17 (21.5%)	60 (77.9%)
< 2 years	104 (17.9%)	493 (85.1%)
Chi-square/*p*-value	0.060/0.805	

### HIV status

Table 4 shows the HIV status of the women surveyed broken down by the demographics and their answers to the survey questions. The only significant difference between the HIV test results was between the age categories (*p* = 0.032). There were no significant differences between and within groups for education (*p* = 0.077), age at first pregnancy (*p* = 0.786), and contraceptive use (*p* = 0.577) as highlighted by the chi-squared analysis (see table).

**Table 4:** Knowledge About Cervical Cancer in Relation to HIV Status

	Positive	Negative
Age		
< 40	75 (25.43)	221 (74.66)
40–50	41 (22.53%)	141 (77.47%)
> =50	29 (15.34%)	160 (84.66%)
Chi-Square/*P*-value	6.863/0.032	
Education		
Did not attend	30 (18.75%)	130 (81.25%)
O′ Level	29 (16.67%)	145 (83.33%)
A’ Level	1 (33.33%)	2 (66.67%)
Grade 7	85 (25.67%)	245 (74.24%)
Chi-Square/*P*-value	6.841/0.077	
Age of First Pregnancy		
< 15	4 (28.5%)	10 (71.43%)
15–20	79 (22.64%)	270 (77.36%)
20–30	39 (19.80%)	158 (80.21%)
> =30	24 (23.07%)	80 (76.92%)
Chi-Square/*P*-value	1.0624/0.786	
Response to: “have you heard of cervical cancer?”		
Yes	115 (21.30%)	425 (78.71%)
No	30 (24.19%)	94 (75.81%)
Chi-Square/*P*-value	0.360/0.548	
Response to: “have you heard of HPV/human papilloma virus”		
Yes	13 (16.67%)	65 (83.33%)
No	132 (22.49%)	455 (77.51%)
Chi-Square/*P*-value	0.459/0.481	
Response to: “please tell me whether you agree or disagree: Cervical cancer can be prevented.”		
Yes	120 (20.87%)	455 (79.13%)
No	4 (20%)	16 (80%)
I don’t know	21 (29.58%)	50 (70.42%)
Chi-Square/*P*-value	0.0089/0.925	
Response to “Against what disease does the HPV vaccine protect?”		
Correct	6 (17.6%)	139 (22%)
Incorrect	28 (82.4%)	491 (78%)
Chi-Square/*P*-value	0.368/0.543	
Have you been Tested for HPV/Cervical cancer?		
Yes	5 (15.15%)	28 (84.85%)
No	140 (22.08%)	494 (77.92%)
Chi-Square/*P*-value	0.885/0.346	
Have you been diagnosed with cervical cancer?		
Yes	1 (12.50%)	7 (87.50%)
No	144 (21.88%)	514 (78.11%)
Chi-Square/*P*-value	0.408/0.522	
What was your age at first sexual intercourse?		
< 15	7 (30.43%)	16 (69.57%)
15–20	80 (22.41%)	277 (77.59%)
20–30	27 (17.53%)	127 (82.47%)
> =30	32 (24.62%)	103 (79.23%)
Chi-Square/*P*-value	3.013/0.389	
Have you ever used oral contraceptive pills?		
Yes	101 (21.09%)	378 (78.92%)
No	40 (23.12%)	133 (76.88%)
Chi-Square/*P*-value	0.310/0.577	
Is your husband/partner circumcised?		
Yes	14 (29.79%)	33 (70.21%)
No	105 (19.02%)	447 (80.98%)
I don’t know	3 (27.27%)	8 (72.73%)
Chi-Square/*P*-value	3.1531/0.0757	

## Discussion

### Knowledge, attitudes, and practices of cervical Cancer and HPV infection

In this study, we evaluated how knowledge and attitudes about cervical cancer and HPV relate to behaviors and actual HPV infection. We found widespread awareness of cervical cancer across age, education, and age of first pregnancy categories. The knowledge that cervical cancer can be prevented was highest among those < 50 years old and those with a grade 7 or secondary education. We postulate that younger individuals are exposed to information about cervical cancer through technology and those who have had advanced schooling benefit from health education taught in schools. This also suggests that at least some of the radio/media campaigns implemented by the Ministry of Health and its agencies have made an impact. Many women reported they had heard about cervical cancer from community health workers. This is important because community health workers were trained on cervical cancer during the pre-study period, so it is possible that some of the knowledge was a byproduct of the study planning period which may have falsely increased the numbers aware of cervical cancer in some communities.

Although we might expect that knowledge of cervical cancer would lead to more positive health behaviors, a common misconception in public health is that knowledge and information drive behavior changes [15]. This may be especially true for women in Zimbabwe where most women still face problems with accessing healthcare both because of the cost and distance from health facilities. Additional reasons may include the social stigma associated with pelvic exams and cancer, and a lack of knowledge about where to get screened. This is compounded by the fact that there are shortages in chemotherapy and treatment machines, making a cancer diagnosis seem like a death sentence. As a result, broad awareness of cervical cancer did not translate into knowledge of HPV or differences in patterns of screening and diagnosis.

### Knowledge, attitudes, and practices of cervical Cancer and HIV infection

Contrary to other prior studies, we did not find a difference in HIV status based on education. Rather, we only found a significant difference between age groups. This is consistent with a prior study of five data sets of Demographic and Health Surveys from Burkina Faso, Cameroon, Ghana, Kenya, Tanzania, where researchers found that education is not positively associated with HIV status. This would not be expected given the positive benefits generally attributed to increased education on health behaviors. The prior study concluded that although school does lead to protective health behaviors, it also predicts a higher level of extramarital sex and lower level of abstinence. These behaviors could cancel each other and could explain in this case why education is not significantly associated with HIV or HPV status [16–18].

Another explanation may exist given the history of education and the HIV epidemic in Zimbabwe. After Zimbabwe gained independence in 1980, the government prioritized providing free primary and secondary education to all. From 1979 to 1984, the number of primary schools in operation increased by 73.3% and the number of secondary schools increased by 537.8% [19]. Zimbabwe aimed to achieve universal education and by the 1990s primary schooling was nearly universal and over half the population had completed secondary education [20]. This expansion of primary and secondary education would most greatly impact those who were of grade school age and high school age during that time – the average age of entering primary school is 5, while for secondary school it is 15. At the time of the survey in 2017, these individuals who would have benefited most from the expansion in education fall into the age 40–50 group and age > =50 group. However, during this time there was also a high death rate among HIV positive women as there was not ready access to ART. The first cases of AIDS in Zimbabwe were the mid-1980s, but it wasn’t until 2002 that the government dedicated resources to scaling up HIV/AIDS treatment. By the time ART became more readily available, educational resources started diminishing. A decrease in GDP starting in the 2000s and continuing to 2008 led to drastic decreases in resources dedicated to social services such as health and education.

This could explain our findings on education, as well as age. Before uptake of anti-retroviral therapy, women died from disease reducing the prevalence in the women surveyed. The incidence of HIV is now decreasing with greater use of ART and more screening programs.

Contraceptive use was also not associated with reduced rates of HPV or HIV in our study. Although contraceptive use is a positive health behavior, the women who use contraceptives might not be using condoms as frequently. Oral contraceptive pills would not have a protective effect against HPV and HIV viruses, and thus this positive health behavior might be cancelled out by less frequent condom usage. One study showed that oral contraceptives have been associated with an increased risk of cervical cancer for women with human papillomavirus infection [21].

## Conclusions

Our study shows that surprisingly, in rural Zimbabwe, age of first pregnancy, education levels, oral contraceptive use, knowledge of cervical cancer and HPV don’t have an association with risk of contracting HPV and HIV overall. The importance of the findings that certain potential risk factors that typically stratify HPV infections are not significant in our study could be due to two factors. While it is possible that the numbers are too small to see a significant difference, it is also possible that HPV infections are more ubiquitous in the community and therefore these risk factors are less significant. This is important because it suggests that rural women have a more uniformly high risk of contracting HPV and should therefore all be preferentially included in screening programs.

Our findings also suggest that the low rates of screening are not due to lack of knowledge alone, rather the lack of available resources for screening. To enable more rural Zimbabwean women to access screening for cervical cancer, one consideration for integrating cervical cancer screening services into existing outreach services is to offer self-collected HR-HPV screening in conjunction with existing outreach for ART and childhood vaccine clinics and/or rural hospital routine care integrated into ART drug administration and/or antenatal care. Extending outreach services, which rural women are already attending, to address cervical cancer education and screening triage would provide an opportunity to bridge the gap between knowledge and screening availability and address some of the barriers to care still affecting women in many regions of the world.

## Data Availability

All de-identified data is available to the public on request.

## Abbreviations

AIDS: Acquired Immunodeficiency Syndrome
ART: Antiretroviral Therapy
DHS: Demographic and Health Survey
EPI: Expanded Program on Immunization
GDP: Gross Domestic Product
HIV: Human Immunodeficiency Virus
HPV: Human Papillomavirus
HR-HPV: High Risk-Human Papillomavirus
LMIC: Low- and middle-income countries
MOHCW: Ministry of Health and Child Welfare
UNAIDS: Joint United Nations Programme on HIV/AIDS
VIAC: Visual Inspection with Acetic Acid plus Cervicography
WHO: World Health Organization
